# Factors Influencing Medicine Use Behavior in Adolescents in Japan Using a Bayesian Network Analysis

**DOI:** 10.3389/fphar.2019.00494

**Published:** 2019-05-07

**Authors:** Chihiro Sakai, Kazuhiro Iguchi, Tomoya Tachi, Yoshihiro Noguchi, Shingo Katsuno, Hitomi Teramachi

**Affiliations:** ^1^Laboratory of Community Pharmacy, Department of Pharmacy, Gifu Pharmaceutical University, Gifu, Japan; ^2^Laboratory of Clinical Pharmacy, Department of Pharmacy, Gifu Pharmaceutical University, Gifu, Japan; ^3^Gifu Pharmaceutical University, Gifu, Japan

**Keywords:** Bayesian, medicine education, medicine use, high schools, Japan

## Abstract

**Background:** Medicine education in Japan was introduced to junior high schools in 2012. However, the effectiveness of existing education programs is limited. In order to develop more effective programs for high school students, the present study investigated the variables that directly influence medicine use behavior and the magnitude of their influence, using a Bayesian network analysis.

**Methods:** A national cross-sectional survey was conducted in 2017. Eighty-three public high schools across Japan were randomly selected, and questionnaires were administered to 15–16 years old 10th grade students. The number of valid responses was 17,437 (effective response rate was 98.46%). Responses were analyzed to measure students’ behavior toward, attitudes regarding, and knowledge of medicines, and awareness of their prior medicine education.

**Results:** Students’ “attitude score” and “awareness of a class” directly influenced their “behavior score.” The “score on attitude,” which had a large influence on “score on behavior,” was directly influenced by “score on knowledge of proper use” and “awareness of class.”

**Conclusion:** The present study argues that acquiring knowledge of appropriate medicine use leads to the acquisition of favorable attitudes, which may result in behavioral change. Therefore, for medicine education, it is expected that incorporating content related to knowledge acquisition for changing attitudes will be important for promoting behavioral change.

## Introduction

As Japan’s society ages, the amount spent on medical care has increased. In 2014, Japan’s medical expenses reached 40.8 trillion yen, exceeding 40% of its national budget (Ministry of Health, Labour and Welfare-Japan, 2016). Therefore, initiatives are needed to reduce the amount government spends on medical care. In recent years, several organizations have proposed self-medication as one way to address this need.

In order to change or promote health-related behaviors, including self-medication, health education is considered a necessary component (Green and Kreuter, 1991). As part of environmental improvements to support self-medication practices, Japan’s Ministry of Health, Labor and Welfare introduced the Revised Pharmaceutical Affairs Law (Ministry of Health, Labour and Welfare-Japan, 2004) in 2006, which greatly changed the system for selling over the counter (OTC) drugs. At the same time, the ministry indicated a need for “medicine education” in schools as a supplementary resolution to the revised Pharmaceutical Affairs Law. As a result, policies regarding environmental improvement and health education for promoting self-medication were introduced across Japan.

Based on revisions to the Pharmaceutical Affairs Law, Japan’s Ministry of Education, Culture, Sports, Science and Technology introduced new content regarding the proper use of medicines in the health and physical education field, specifically the “New Course of Study for Junior High School Students” (Ministry of Education, Culture, Sports, Science and Technology-Japan, 2006). As a result, medicine education has been implemented in classes on health through physical education departments in junior high schools since 2012.

From November 2014 to January 2015, after the commencement of the aforementioned medicine education, Teramachi et al. (2012) surveyed the implementation status of this provision throughout Japan, reporting that nearly all junior high schools offered medicine education classes. However, when Teramachi et al. (2016) surveyed students in their 1st year of high school within the Gifu prefecture, only 31% remembered going through the curriculum. About 70% responded that they either never took a medicine education class or did not remember doing so. These findings suggested that the effectiveness of the current approach might be limited. Thus, in order to promote students’ proper medication use behavior, it is necessary to develop more effective teaching methods, content guides, and programs.

Based on the PRECEDE-PROCEED model proposed by Green and Kreuter (1991) various factors, such as predisposing factors (attitudes, knowledge, and beliefs), enabling factors (skills), and reinforcing factors (support from surrounding people), are involved in health behavior. Therefore, to promote desired behaviors, it is necessary to address relevant and inter-related factors through health education. Conventional research relies on statistical methods (such as univariate or multivariate analyses) to examine the relationship between the objective variable (behavior) and the explanatory variable (factors affecting behavior). However, the PRECEDE-PROCEED model demonstrates that the mechanisms governing a person’s behavior are complicated, and while a conventional statistical method can clarify the relationship between the objective variable and the explanatory variable, this method has limited ability in clarifying the relationship between various explanatory variables.

In recent years, Bayesian network analyses, which are based on Bayes’ theorem and advocated by Pearl (1985), have drawn attention. This method makes it possible to visually represent complex causal relationships among multiple variables. The application range of a Bayesian network analysis is wide, and this analysis can be used as a form of factorial analysis in various research areas. A key benefit is that this method makes it possible to clarify not only the relationship between objective and explanatory variables but also the relationship between explanatory variables in a complex and causal manner. Thus, Bayesian analyses are better suited for understanding the complicated structures and mechanisms underlying health behavior.

Previous research has reported several variables relating to adolescent medicine use. Adolescent medicine use behavior is influenced by age, (Morales-Suarez-Varela et al., 2009) gender, (Chambers et al., 1997; Hansen et al., 2003; Bulck et al., 2005; Andersen et al., 2006; Due et al., 2007) attitudes, (Sakai et al., 2014) self-evaluation of health conditions, (Tobi et al., 2003; Holstein et al., 2008b) length of time watching TV, (Bulck et al., 2005; Morales-Suarez-Varela et al., 2009) ease of obtaining medicine, (Holstein et al., 2008a), and socio-economic situation (Holstein et al., 2004). Here, a Bayesian network analysis is a more useful means for clarifying the structure of adolescent medicine use behavior given that multiple variables influence each other in complicated ways. Previous studies have used conventional univariate or multivariate analyses, but none have examined the relationship between variables using Bayesian networks.

In order to obtain basic data for developing an educational program for promoting proper medicine use behavior among high school students, we examined variables likely to have a direct influence on medicine use behavior, as well as the magnitude of such influence, using a Bayesian network method.

## Materials and Methods

### Participants

The target population included 10th grade high school students (15–16 years old, both boys and girls). In principle, all the students belonging to the grade participated in this study. The number of questionnaires distributed was 17,895 at 81 schools; 17,812 were collected, with 17,709 responses received. A total of 272 incomplete responses were excluded (e.g., did not answer the latter half of the questionnaire and/or did not disclose gender). There were 17,437 (8,205 male and 9,232 female students) valid responses received, and the effective response rate, the number of valid response divided by the number of people who answered the survey, was 98.46%.

### Instruments

Data were collected using an anonymous self-administered questionnaire comprising 13 items. The contents of the questionnaire were examined by school teacher to determine whether students could understand them or not. Additionally, this questionnaire had been used in our previous studies (Teramachi et al., 2012, 2016). The following explanation was stated at the beginning of the questionnaire: ‘Please tell me what you think about medicine. “Medicine” used in this questionnaire refers to the medicine you get at the hospital or buy at a community pharmacy. It includes not only medicines for internal use but also compresses, external medicines, and disinfectant used for injuries and other occasions. It also includes household medicines, eye drops, troches, and inhalants. However, it does not include nutritional supplements or energy drinks. Please do not write anything if you do not want to answer the questionnaire. Not completing the questionnaire will not affect your grades.’ Students answered the questionnaires in their classrooms after receiving standardized instructions. Homeroom teachers typically distributed the questionnaires. At the beginning of the questionnaire, it was noted that the questionnaire was not a requirement, and a student could stop at any time. In addition, completing the questionnaire was treated as consent to participate. The Gifu Pharmaceutical University ethics committee confirmed that this study was conducted in accordance with the ethical principles that have their origins in the Declaration of Helsinki, and approved the protocol. The ethical committee approval included the principal’s written informed consent in lieu of parental consent, and deeming the answer to the questionnaire as consent by the students themselves.

Healthcare and medicine use were assessed through the following questions: (1) What do you do when you are in poor physical condition? (i.e., go to sleep early, take medicine at home, consult with families, consult with a teacher, see a doctor, consult a pharmacy, other); (2) For what purpose do you use medicine? (i.e., stomachache, headache, cold, fever, toothache, allergies, car sickness, other); (3) Who do you consult when you use medicine? (i.e., parents/grandparents, brothers/sisters, friend, doctor/dentist, pharmacist, school teacher, I have medicines that I take regularly, there is no medicine I take regularly, other); and (4) Have you ever done the following: purchased medicine using your own judgment, received medicine from a friend, gave medicine to a friend?

Behavior regarding, attitudes toward, and knowledge of medicine were assessed through the following questions: (1) When you use medicine, what kinds of things are you careful about? (i.e., read the description, check the dosage, check the dosage time, check that I had a meal, take medicine with water, ensure the medicine agrees with my constitution, I do not care, other); (2) When you use medicine, what do you think is important to be careful of? (i.e., read the description, check the dosage, check the dosage time, check that I had a meal, take medicine with water, ensure the medicine agrees with my constitution, other); (3) What terminology do you know? (i.e., OTC medicine, medical medicine, generic medicine, family pharmacy, medication notebooks, doping, school pharmacist); and (4) Which items related to a medicine’s proper use do you know? (i.e., do not take medicine with milk or juice; do not bite tablets or disassemble capsules; between meals is not the same as during meals; take medication for the indicated number of days; most medicines have some side effects; do not overdose even if the medicine does not work soon; do not double the dosage, even if you forget to take it once; cold OTC medicine is symptomatic treatment). Respondents were asked to select “yes” for all choices that applied to them on each of the lists.

To determine students’ experience with medicine education, respondents were asked whether they had ever taken classes on medicine in school. We asked respondents who remembered taking medicine education classes an additional question addressing under which subject they took the classes. Finally, students were asked to record their gender.

### Procedures

A cross-sectional, school-based survey was conducted across Japan from May to July 2017. Two or three public high schools from each prefecture were selected by simple random sampling method using a 2016 list of all high schools in Japan. Prior to the survey, the purpose and protocol of this research were explained to the education committee of all prefectures and Japan’s Ministry of Education, Culture, Sports, Science and Technology by mail. The purpose of the survey was explained to the principal of each school by mail, and written consent was received. If the proposal was declined, we asked another school in the same area. We obtained initial consent from 83 school principals. Our final dataset included surveys from only 81 schools; two were excluded from the analyses because the schools conducted the questionnaire in August, which fell outside the target period.

Although the first medicine education class is always carried out in 9th grade (i.e., junior high), the timing of advanced curriculum varies between high schools. Therefore, we distributed our questionnaires to students in 10th grade, who were 15–16 years old, and in their 1st year of high school (from May to July), shortly after they began the school year.

### Statistical Analyses

Questions regarding behaviors, attitudes, knowledge of terminology, and knowledge as to proper medication use were scored for each item. The total score for each item was calculated with the answer “Yes” as 1 point. Furthermore, in the Bayesian network, since it is necessary to discretize continuous data, items were categorized into three groups (low, medium, and high). Specifically, for behavior: 0 to 2 points were categorized as low, 3 to 5 points as medium, and 6 to 8 points as high. For attitudes: 0 to 2 points were deemed low, 3 to 4 points as medium, and 5 to 6 points as high. For knowledge: a score of 0 to 2 points was categorized as low, 3 to 5 points as medium, and 6 to 7 points as high. Finally, for knowledge on proper use of medicines: 0 to 2 points were deemed low, 3 to 5 points as medium, and 6 to 8 points as high.

The purpose of this study was to clarify variables that directly influence the “behavior score” and to examine the relationship between the variables; the concrete analytic procedure is as follows:

First, to visualize the relationship between variables, we constructed the model using all questionnaire items.

Next, we verified the constructed model.

Then, a sensitivity analysis was performed to clarify the magnitude of the influence on the “behavior score” for each variable.

Fourth, we constructed evidential reasoning by giving conditions to the “attitude score” and “awareness of a class,” which directly affected the “behavior score.”

Finally, we constructed evidential reasoning by giving conditions to “knowledge of proper use” and “awareness of a class,” which directly affected the “attitude score,” which had a larger influence on the “behavior score.”

As no variables were related to “gender” and “awareness of a class,” these two variables were set as having no patent nodes. As per our methodology, it was necessary to use different data for constructing the model and for verifying the model, with a preferred ratio of 4 to 1. Therefore, we divided the 17,437 respondents into this approximate ratio. Thus, data from 14,000 participants was used to construct the model, and data from 3,437 participants was used to verify the model.

BayoLink version 7.1 software, developed by NTT DATA Mathematical Systems Inc., Tokyo, Japan, was used for building and reasoning the Bayesian networks. The causal relationship (connection method between nodes) among Bayesian networks was determined by a search algorithm using a greedy strategy, and Akaike’s Information Criterion (AIC; Akaike, 1973) was adopted as the evaluation criterion.

## Results

### Establishment of the Medicine Use Behavior Model

**Figure 1:**
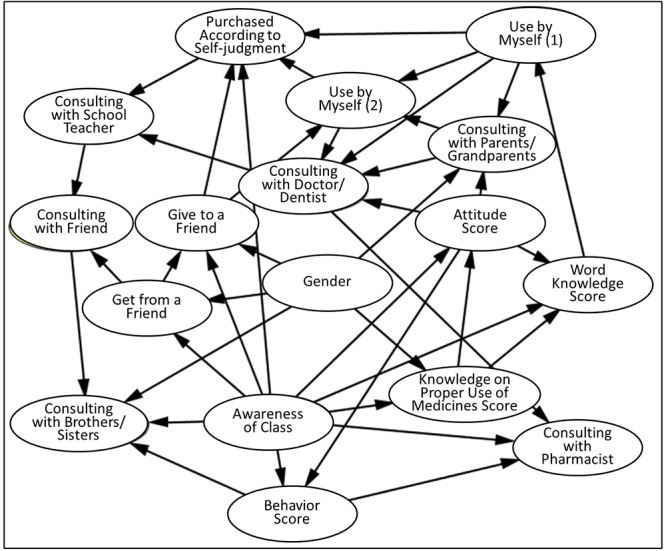
The constructed model of students’ medicine use behavior.

The constructed model is shown in Figure 1, and the observed value of each node is shown in Table 1. The constructed model showed that two variables—“attitude score” and “awareness of a class”—were directly related to the “behavior score.” The “attitude score” was directly related to the “knowledge of proper use score” and “awareness of a class.”

**Table 1:** Observed value of each node.

Node (State)	Observed value
Gender	
Male	0.4702
Female	0.5298
Consulting with Parents/Grandparents	
Yes	0.863
No	0.137
Consulting with Brothers/Sisters	
Yes	0.0262
No	0.9738
Consulting with Friend	
Yes	0.0221
No	0.9779
Consulting with Doctor/Dentist	
Yes	0.2495
Ne	0.7505
Consulting with Pharmacist	
Yes	0.0673
No	0.9327
Consulting with School Teacher	
Yes	0.0128
No	0.9872
Use by Myself (1)^a^	
Yes	0.1279
No	0.8721
Use by Myself (2)^b^	
Yes	0.1672
No	0.8328
Purchased due to Self-judgment	
Yes	0.1239
No	0.8761
Get from a Friend	
Yes	0.1378
No	0.8622
Give to a Friend	
Yes	0.1427
No	0.8573
Score on Behavior	
High	0.0442
Middle	0.6185
Low	0.3374
Score on Attitude	
High	0.3285
Middle	0.3833
Low	0.2882
Score on Word Knowledge	
High	0.3142
Middle	0.5286
Low	0.1572
Score on Knowledge on Proper Use of Medicines	
High	0.4651
Middle	0.4163
Low	0.1186

^a^*(1)There are medicines that are to be used regularly; ^*b*^(2) there is no medicine that is to be used regularly*.

### Evaluation of the Model

The medicine use behavior model was verified from 3,437 responses. The model was evaluated using the correct data number (the number correctly checked against results from inference and verification data), correct answer rate (what percentage of all data gave the same result as the model), relevance rate (among data predicted via reasoning: the verification to prediction ratio), recall rate (among verification data values: the ratio at which verification matched prediction), and F-Measure (the harmonic mean of the relevance and recall rate). The F-measure is an index of performance evaluation proposed by Kitamura et al. (2005) where the closer to 1 (in a 0 to 1 range) the value is, the better the model performs.

The number of correct answers was 2,547, and the correct answer rate was 0.7411. Details of the verification results are shown in Table 2. Both the recall and F-Measures were high within the medium and high groups (and low for the low group) for the “behavior score.”

**Table 2:** Evaluation of the model.

State	Relevance rate	Recall rate	F-Measure
High	1.000	0.2318	0.1647
Medium	0.7563	0.8534	0.6716
Low	0.7079	0.6389	0.6716

### Sensitivity Analysis

**Figure 2:**
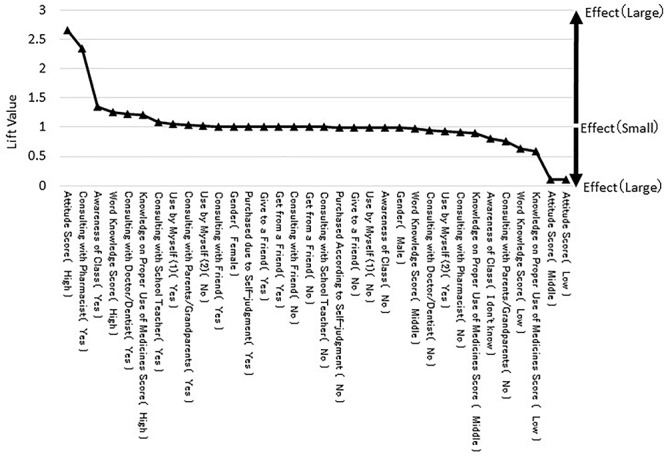
Results of the sensitivity analysis when “behavior score” is “high.”

A sensitivity analysis was performed to quantitatively calculate the magnitude of each variable’s influence on the “behavior score.” Results of the sensitivity analysis when the “behavior score” was “high” are shown in Figure 2. This graph indicates that the influence when the “behavior score” was “high” is small, indicating that sensitivity is closer to 1, and the influence increases as the value deviates from 1. As can be seen in Figure 2, the “attitude score” had the largest lift value. Variables with lift values larger than 1 were “consulting with a pharmacist,” “consulting a doctor/dentist,” “awareness of a class,” “word knowledge,” and “knowledge on proper use.” These variables had a positive influence on a higher “behavior score.” Conversely, “not consulting with parents and grandparents,” “not knowing whether a class was given,” and a low score on “word knowledge,” and “knowledge on proper use,” had negative influences.

### Analysis of Medicine Use Behavior Using the Model

#### Analysis of Variables Directly Related to the Score on Behavior

The model in Figure 1 showed that the “attitude score” and “awareness of a class” directly affected the “behavior score.” In order to analyze the magnitude of these variables’ influence on the “behavior score,” we set up the following three cases and made inferences to examine changes in the “behavior score” probabilities: (1) When conditional on “awareness of a class,” (2) When conditional on the “attitude score,” and (3) When conditional on both “awareness of class “and the “attitude score.”

**Figure 3:**
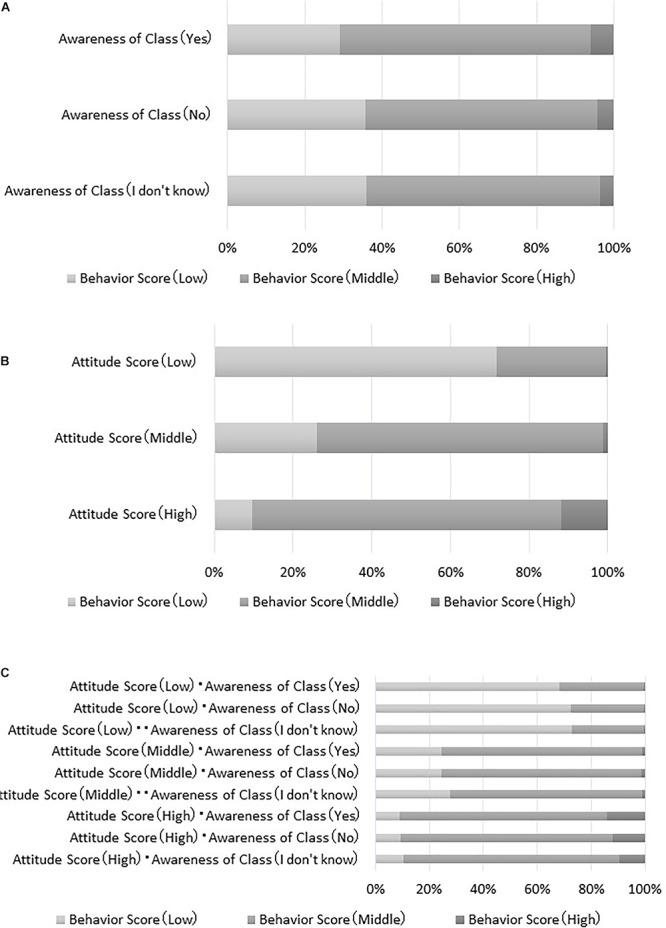
**(A)** Results of “awareness of a class.” **(B)** Results of “attitude score.” **(C)** Results for both “awareness of a class” and “attitude score.”

Figure 3A shows results based on “awareness of a class.” This graph indicates the probability that the “behavior score” will be high, medium, and low as a function of variation in “awareness of a class.” For example, a student who answers “yes” to “classroom experience” will be placed in the “high group” regarding their “behavior score,” with a probability of about 5%. In other words, results in Figure 3A reflect the probability that the “behavior score” will be “high” if answering, “yes,” on the “awareness of a class” as compared to when answering, “no” or “I don’t know.” However, even if a student answers, “yes,” to “awareness of a class,” the probability that the “behavior score” will be “high” is not robust. Here, the probability of the “behavior score” will be “low” is about 40%. These results demonstrate that it is not possible to raise the “behavior score” by only changing “awareness of a class.”

Figure 3B shows results from the “attitude score.” This graph shows the probability that the “behavior score” will be high, medium, and low when the “attitude score” changes. When the “attitude score” is “low,” the probability that “behavior score” will be “low” is relatively high; conversely, the probability that the “behavior score” will be “high” is extremely low. On the other hand, when the “attitude score” is “high,” the probability that the “behavior score” will be “medium” or “high” is also high.

Furthermore, results of both “awareness of a class” and the “attitude score” in combination are shown in Figure 3C. Here, the probability that the “behavior score” is “high” is at its highest when the “attitude score” is “high” and “yes” is answered for “awareness of a class.” The probability that the “behavior score” is “low” is at its lowest when the “attitude score” is “low” and participants answer, “I don’t know,” for “awareness of a class.”

#### Analysis of Variables Indirectly Related to the Behavior Score

Based on the results presented in Figure 2, 3, variables affecting the “attitude score,” which has the greatest influence on the “behavior score,” were analyzed. From the model in Figure 1, the “attitude score” has a direct relationship on the “awareness of a class” and the “knowledge on proper use score.” In order to analyze the magnitude of influence regarding the “attitude score,” the following three cases were set up, and inferences were made to examine the change in probability for the “attitude score”: (1) When conditional to “awareness of a class,” (2) When conditional to the “knowledge on proper use score,” (3) When conditional to both “awareness of a class” and the “knowledge on proper use score.”

**Figure 4:**
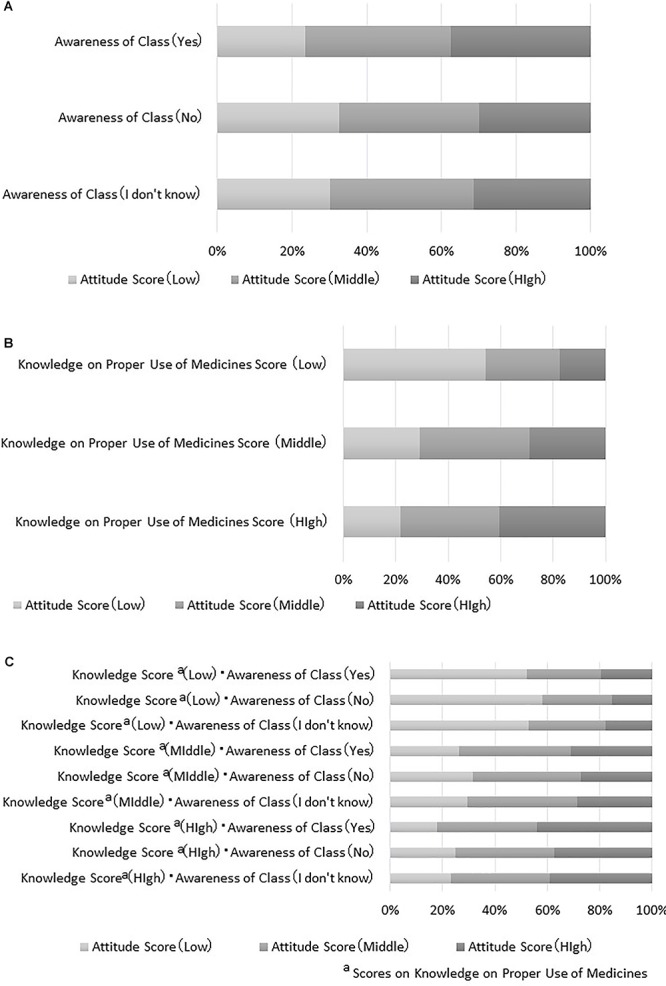
**(A)** Results of “awareness of a class.” **(B)** Results of “knowledge on proper use of medicines score.” **(C)** Results for both “awareness of a class” and “knowledge on proper use of medicines score.”

Figure 4A shows results based on “awareness of a class.” The graph indicates that the probability that the “attitude score” is “high” is slightly higher when participants respond, “yes,” to “awareness of a class” than when answering, “no” or “I don’t know,” to “class experience.” However, the probability did not differ greatly in terms of “awareness of a class.”

Figure 4B shows results based on the “knowledge of proper use score.” The probability that the “attitude score” is “high” reaches the highest when the “knowledge of proper use score” is “high.” The probability that the “attitude score” is “low” reaches the lowest when the “knowledge of proper use score” is “low.”

Results for the combination of “awareness of a class” and the “knowledge of proper use score” are shown in Figure 4C. These results indicate that the probability that the “attitude score” is “high” reaches the highest when the “knowledge of proper use score” is “high” and participants respond, “yes,” to “awareness of a class.” The probability that the “attitude score” is “low” reaches the lowest when the “knowledge of proper use score” is “low” and participants respond, “I don’t know,” to “awareness of a class.”

## Discussion

The present study included a questionnaire-based survey to collect information for developing a medicine education program that effectively brings about appropriate medicine use behavior. This was done by revealing variables that have the greatest impact on proper medicine use behavior. By using a Bayesian network, the present study sought to clarify the complex and causal relationships between medicine use behavior and its related variables (including relationships between related variables). Furthermore, we examined the influence of these variables on behavior by providing conditions whereby these factors could have a significant influence on expected behavior.

In terms of verifying the model, the recall rate and F-measure were large when the “behavior score” was “medium” or “low.” These results indicate that it is better to infer that the “behavior score” is moderate or low than to infer a high score. This is because the number of students in the “high” group from the observation data was 4.5%, which was smaller than the other groups.

Regarding the sensitivity analysis, the absolute lift value for the “attitude score” was the largest. This means that the “attitude score” had the greatest influence on the “behavior score.” More specifically, this result indicates that when the “attitude score” was high, the “behavior score” increased. However, when the “attitude score” was low or moderate, the “behavior score” decreased. Therefore, it would appear that improving the attitude score is the most important factor for increasing medicine use behaviors.

It was further revealed that the “attitude score” and “awareness of a class” directly influenced the “behavior score.” Additionally, by inferring how much the “behavior score” changes when conditions are given to the “attitude score” and “awareness of a class” variables, it became clear that increasing the “attitude score” contributes greatly to increasing the probability of engaging in more appropriate medicine use behavior than whether or not students had a medicine education class. This provides further evidence that attitudes are the most influential component to medicine behavior change.

Interestingly, the “attitude score” was directly influenced by the “knowledge of proper use score” and “awareness of a class.” As a result of inferring how much the “attitude score” would change based on conditional aspects of the “knowledge of proper use score” and “awareness of a class,” it became clear that increasing the “knowledge of proper use score” greatly contributed to increases in the probability of holding more favorable medicine use attitudes than whether the student had taken a class on medicine use. Thus, it appears that acquiring knowledge regarding the proper use of medicines leads to the acquisition of favorable attitudes, which may result in behavioral change. Therefore, for medicine education, incorporating content related to knowledge acquisition for engendering attitude change should be important for promoting behavioral change. Furthermore, prior exposure to medicine education classes alone, does not seem sufficient for influencing behaviors or attitudes.

Our finding that attitudes had the greatest influence on medicine use behavior has been previously demonstrated in a prior study (Sakai et al., 2014). According to the results of multiple logistic regression analysis on the survey of 1,369 high school students in Japan, attitude was a more important factor associated with students’ medicine use behavior than stress coping skills, stress response, family-related self-esteem, and smoking (Sakai et al., 2014). The present results could also be reasonably explained through existing behavioral science theories. For example, the Theory of Planned Behavior proposed by Ajzen (1991) posits that three factors influence “behavioral intentions”: “subjective norms” (an individual’s perception about the particular behavior, which is influenced by one’s social context), “perceived behavioral control” (an individual’s perceived ease or difficulty with performing the particular behavior), and “attitude toward behavior” (the degree to which a person has a favorable or unfavorable evaluation of the behavior of interest). Our results, while obtained from a model constructed using a Bayesian network, support the notion that behavior is directly and largely influenced by attitudes, specifically attitudes acquired through knowledge.

Although “consulting with a pharmacist” and “consulting a doctor/dentist” did not have a direct influence on the “behavior score” in the constructed model, the sensitivity analysis revealed that these variables had some role in the “behavior score.” From the PRECEDE-PROCEED model (Green and Kreuter, 1991), it is known that not only predisposing factors (such as attitudes and knowledge) but also the presence of reinforcement staff (such as support from surrounding others) is important for behavior change. Therefore, it may be necessary not only to support the acquisition of correct knowledge and favorable attitudes at school but also to make it easier to obtain support from professionals in order to promote appropriate medicine use behavior.

The present study is the largest survey of medicine use behavior among Japanese students. It is also the only study to investigate the relationship between medicine use behavior and related variables using Bayesian networks. Our methodology is robust (random extraction of surveyed schools from all regions in Japan, a short survey period in order to minimize bias, etc.). The questionnaire we used is reliable and valid, as it was modified from a prior questionnaire used for targeting 5,612 elementary, junior high, and high school students nationwide, as well as 1,399 high school students within the Gifu prefecture (Teramachi et al., 2012, 2016). The results of Table 1, representing descriptive statistics of each question, were quite similar to the results of our previous survey (Teramachi et al., 2012, 2016). This implies that the questionnaire which we used has reliability and validity, and that it may be possible to generalize the results of this study and previous our studies to some extent. Nevertheless, there is a possibility of self-selection bias within our participant sample. However, our effective response rate was 98.46%, and the main reason for invalid responses was failure to complete the second half of the questionnaire or neglecting to disclose one’s gender.

A few other limitations should be noted. First, there is a possibility that respondents demonstrated a recall bias. Second, given that we implemented a questionnaire survey, a limited amount of information could be collected. While we asked questions regarding medicine use behavior, as well as attitudes, knowledge, and medication use experience, there may be other important variables that affect medicine use behavior. Third, there are few studies which focused on factors influencing adolescents’ medicine use behavior and which used Bayesian network analysis, it was difficult to compare the results of this study with previous studies. Fourth, simple random sampling method was used to select highs schools. Although high schools in all region were included in this study, the population of each region was not considered.

Due to revisions in education guidelines, medicine education was recently introduced to junior high schools throughout Japan. However, there is currently no evidence based medicine education program that has gathered implementation success in Japan. In order to effectively promote healthy medicine use behaviors through school education, it is necessary to clarify the variables that influence these behaviors and consider providing clear guidance as to the content and instruction methods necessary to deliver desirable outcomes.

## Ethics Statement

The Gifu Pharmaceutical University ethics committee approved the protocol. The purpose of the survey was explained to the principal of each school, and written consent was received. Students answered the questionnaires in their classrooms after receiving standardized instructions. Homeroom teachers typically distributed the questionnaires. At the beginning of the questionnaire, it was noted that the questionnaire was not a requirement, and a student could stop at any time. In addition, completing the questionnaire was treated as consent to participate.

## Author Contributions

All the authors provided substantial contributions to the development of the manuscript. HT and SK contributed to the conception and design of the study, data acquisition, and revising the manuscript. KI, TT, and YN contributed to the analysis and/or interpretation of the data, drafting the manuscript, and revising the manuscript. CS contributed to the analysis and/or interpretation of the data and drafting the manuscript.

## Conflict of Interest Statement

The authors declare that the research was conducted in the absence of any commercial or financial relationships that could be construed as a potential conflict of interest.
